# A health promotion intervention to improve oral health of prisoners: results from a pilot study

**DOI:** 10.1108/IJPH-11-2020-0085

**Published:** 2021-07-16

**Authors:** Kjersti Berge Evensen, Vibeke Hervik Bull, Linda Ness

**Affiliations:** Oral Health Centre of Expertise-Rogaland, Stavanger, Norway

**Keywords:** Prisoners, Prison, Oral hygiene, Oral health, Motivational interviewing (MI), Oral health-related behavior

## Abstract

**Purpose:**

Prisoners have poorer oral health than the general population. Good oral health is essential for both social and physical well-being. For prisoners, poor oral health is also related to drug use after release, whereas good oral health is related to successful reintegration into society. The purpose of this study was twofold: to examine the effect of an intervention based on motivational interviewing (MI) on prisoners’ oral health-related behavior and to assess if the intervention is a good fit for this population.

**Design/methodology/approach:**

In total, 16 prisoners in a Norwegian prison were offered a brief MI-based intervention focusing on changing their oral health-related behavior. An oral examination was also performed and the prisoners received a small package containing oral hygiene aids. Two weeks later, a second oral examination and a semi-structured interview were conducted to explore the effect of the intervention and examine the prisoners’ responses to the intervention. Qualitative data analyzes were guided by thematic analysis.

**Findings:**

The findings indicate that the intervention had positive effects on both the prisoners’ motivation to use oral health-related behavior and their performance of oral health-related behavior. The findings also indicate that the intervention was well adapted to the target population.

**Originality/value:**

This is one of the first studies that explore the effect of an intervention in improving prisoners’ oral health and bridges a knowledge gap in the literature. The findings may increase the understanding of how dental services should be organized and offered to provide dental health care to this vulnerable group.

## Introduction

Little is known about prisoners’ oral health in Norway. To the best of our knowledge, only one study on prisoners and oral health has been conducted in the country, examining hepatitis B serum markers and oral health in a group of male prisoners (Hurlen *et al.*, 1984). The only overview of prisoners’ oral health status in Norway can be found in a national report on prisoners’ living conditions (Revold, 2015). Oral health is mentioned only briefly in this report, but it states that prisoners in Norway have significantly poorer dental health than the rest of the population. A total of 37% of prisoners consider their dental health poor or rather poor compared to 6% of the general population. Also, 34% report that they have needed to visit the dentist in the past year but have not done so. This is much higher than for the rest of the population (14%; Revold, 2015).

This is in line with findings from studies conducted in other countries on prisoners’ oral health. Compared to the general population, prisoners are more likely to exhibit a higher degree of oral disease, have not received dental treatment regularly and have less motivation and capability to maintain their oral health (Guarnizo-Herreño *et al.*, 2014; Heidari *et al.*, 2007; Jones *et al.*, 2002; Osborn *et al.*, 2013; Rodrigues *et al.*, 2014; Vainionpää *et al.*, 2017). They also have more missing and decayed teeth and fewer filled teeth compared to the non-prison population (Akaji and Ashiwaju, 2014). The reasons for prisoners’ poor oral health are complex, but as a group, they are more likely to have disadvantaged backgrounds in terms of low socioeconomic status, higher rates of unemployment, lack of education and greater experience of trauma or abuse when compared to the non-prison population (Donnelly *et al.*, 2019; Walsh *et al.*, 2008). People from poor socioeconomic backgrounds have more difficulties in accessing health-care services (Cinar, 2016; Cohen, 1987; Freeman and Richards, 2019) because of low understanding of health and health-care services, low perception of need or costs of treatment (Donelle and Hall, 2014; Donnelly *et al.*, 2019; Freeman and Richards, 2019). Also, prisoners are more likely to practice health-damaging behaviors – involving poor diet and high consumption of tobacco, alcohol and illicit substances – compared to the general population, which also contributes to their poor health (Arora *et al.*, 2020; Vainionpää *et al.*, 2017).

Improving prisoners’ oral health is important for many reasons. Good oral health has a significant impact on quality of life and is essential for both general health and social well-being (Slade, 1997). A study by Arora *et al.* (2020) found that prisoners’ poor oral health influenced their general well-being, especially in terms of mouth-related pain. The study also reported that prisoners’ poor oral health had an impact on their psychological discomfort and disability in terms of feeling self-conscious and embarrassed about the appearance of their teeth. For prisoners, good oral health status is closely related to successful reintegration into the outside world (Janssen *et al.*, 2017). Janssen *et al.* (2017) found that prisoners with a toothache on release from prison had an increased risk of using opioids after release and returning to criminal activity. The study also indicated that having missing or damaged teeth made it more difficult for them to find a place to live or get a job after release. If good oral health care is provided to prisoners, it will probably be beneficial to them as individuals, their families and the community (Treadwell and Formicola, 2005).

The World Health Organization emphasizes the importance of all people having access to health-care services that are provided in a way that meets individual preferences and needs (Cinar, 2016). This means that prisoners should be offered a prison environment that encourages health and that health-promoting programs should be specifically designed for individual prisoners (Cinar, 2016). However, little is known about which interventions are effective in improving prisoners’ oral health and how such interventions should be adapted to them individually (Forsberg *et al.*, 2011).

On the basis of this knowledge gap concerning prisoners’ oral health and how to improve it through individually adapted interventions, this study’s aim was twofold: (i) develop and test an intervention to improve prisoners’ oral health and (ii) assess how well the intervention fits the population of interest.

To develop an oral health-promoting intervention, it was essential to find a method that was both effective in improving oral health and capable of adapting to this population in terms of meeting their specific individual health preferences and needs. Motivational interviewing (MI; Miller and Rollnick, 2002) was considered to meet these criteria. MI is an individually oriented, goal-directed therapeutic method that helps people resolve ambivalence about health behavior by strengthening their motivation and commitment to change (Miller and Rollnick, 2002). Several studies have found it to be useful in improving oral health-related behavior (Cascaes *et al.*, 2014; Godard *et al.*, 2011; Harrison *et al.*, 2007; Kay *et al.*, 2016). MI is also a therapeutic intervention considered particularly useful for groups of clients that normally do not respond to traditional therapeutic interventions (Rubak *et al.*, 2005). Relatively few studies have been conducted to examine the effect of MI in correctional settings and a literature review found that findings from these studies are mixed regarding the effect of MI  (McMurran, 2009). Antiss *et al.* (2011) found that prisoners who were offered MI showed an increased readiness to change their behavior in the period of recovery. Forsberg *et al.* (2011) found that prisoners who received MI in prison showed reduced problematic alcohol- and drug-related behavior after their release. Other studies, however, have found no effect of MI on this group (McMurran, 2009). To the best of our knowledge, no studies have examined the effect of MI on improving prisoners’ oral health.

## Method

### Ethical concerns

The study was approved by the Regional Committee for Medical and Health Research Ethics in Western Norway. Both written and verbal information about the intervention and interviews were provided by both the prison health-care service and dental hygienists. To safeguard the anonymity of participants, findings are presented without details that could be used to identify individuals.

### Participants and research site

This study was conducted at the largest prison in the region. It is classified as a high-security prison and has facilities for 68 prisoners, including a section for female prisoners. The participants were both long- and short-stay prisoners, but details of their sentences were not available to the research group.

All detainees in the prison during the period October–December 2018 were asked to participate. They were recruited by the nurse in the prison health-care service on their admission to the prison and were informed about the project, steps to preserve their anonymity and their right to withdraw from the study. They were given both written and verbal information about the project. In total, 24 detainees agreed to participate in the study. Seven of them were however released from the prison or transferred to other prisons before the research interview and one withdrew from the study, leaving 16 participants (14 men and two women).

### The intervention: Motivational interviewing and oral examination

The intervention consisted of an oral examination, one MI-based intervention and a prophylaxis package containing various hygiene aids: toothbrush, toothpaste, toothpicks and dental floss. Two weeks after the intervention, a second oral examination and a research interview were conducted to measure the potential short-term effects of the intervention and assess whether or not the intervention was well adapted to the target population.

The first oral examination was conducted prior to the MI-based intervention. Both the oral examinations and MI-based intervention were performed in a visitors’ room in the prison; the dental hygienist only had a mouth mirror and headlight at their disposal during the examinations. The sparse use of dental tools was regulated by the prison for safety reasons. This first oral examination provided a rough indication of the prisoners’ oral health status. Caries, erosions and missing teeth were assessed following regular clinical standards. The dental hygienist further assessed oral hygiene using the mucosal-plaque index (MPS) (Henriksen *et al.*, 1999). MPS is a standardized, fast and non-invasive method that gives a rough estimate of oral hygiene status. It assesses oral hygiene by measuring levels of mucus and plaque on scales ranging from 1 to 4, respectively. The total MPS score is the sum of both scales. A score of 2–4 is considered good or adequate oral hygiene; 5–6, unacceptable oral hygiene; and 7–8, poor oral hygiene. To ensure calibration of oral examinations across dental hygienists, the hygienists involved in the project conducted several oral examinations on a number of regular patients prior to the study to compare assessments and conclusions of the oral examinations.

In addition to assessing oral health status and hygiene, the first oral examination had the potential to inform the focus of the MI-based intervention. It also allowed the dental hygienist to detect unmet dental treatment needs and refer the prisoners swiftly for dental treatment.

The oral examination was followed by the MI-based intervention. This intervention was developed in close cooperation with two regional competence centers having extensive experience with MI as a methodology for individuals with issues concerning drug and alcohol abuse: (i) KoRus-Vest and (ii) KORFOR.

*KoRus* is one of the seven regional drug and alcohol competency centers in Norway, whereas *KORFOR* conducts research on individuals suffering from substance abuse. The Norwegian Correctional Service was also involved in the development of the intervention, especially in terms of how to adapt it to the prisoners and correctional settings.

The dental hygienists involved in the project attended a three-day training program in MI held by KoRus-Vest. They conducted several motivational interviews in a given period prior to the project and received personal feedback on their performance and coaching from experienced personnel from KORFOR. The intervention applied in this study was further tested on a number of former prisoners. Their responses and feedback were considered and used in the last stage of the intervention development to ensure the intervention was properly adapted to the target population (Evans *et al.*, 2019; Stirman, 2012).

The intervention applied in this study was an adaptation of MI that built upon the MI techniques *open questions*, *affirmations* and *summary reflections*. Together with *reflective listening*, these three techniques are the basic techniques used in the MI approach (Miller and Rollnick, 2002). Reflective listening was, however, excluded from this intervention for two reasons: (i) it demands extensive training to perform this technique successfully and (ii) it requires quite complex mental processes to benefit from this technique (Miller and Rollnick, 2002).

Therefore, to make the intervention easy to perform successfully and increase the likelihood of adopting the intervention to the individual prisoners’ oral health–related needs and cognitive abilities, *reflective listening* was replaced with a *change plan* (Magill *et al.*, 2010). A change plan is a written plan that identifies concrete individual goals for behavioral changes (Magill *et al.*, 2010; Miller and Moyers, 2006).

The primary focus of the MI intervention was the prisoners’ view of their current oral health and the oral health–related behavior they wanted to change. The intervention started with *open questions*, which in MI are used to encourage clients to tell their story in their own words, without leading them in a specific direction. In this phase, the prisoners were encouraged to talk about their own oral health. The dental hygienist asked open questions such as “How do you consider your oral health?” “How often do you brush your teeth?” and “How do you consider your dietary habits.” Open questions were followed by *affirmations*, which are statements aimed at recognizing clients’ strengths and acknowledging behaviors that lead in the direction of positive change. The dental hygienist in this phase confirmed the prisoners’ focus and emphasized support and understanding: “You are trying hard to take good care of your teeth […]. You have tried and succeeded so many times before […]. You have an enormous strength […].” In standard MI interventions, affirmations are followed by *reflective listening*. In this study, the *change plan* was the next stage. It was a written plan with *one* described, concrete goal aimed at improving the individual prisoner’s oral health or related behavior. The plan followed a given structure. First, the target behavior was identified. The identification of the target behavior was based on either findings from the oral examination (e.g. poor hygiene or teeth erosions) or information provided by the prisoners in the *open questions* stage (e.g. not brushing teeth on a regular, daily basis or smoking). One specific target behavior was selected and written down (e.g. brushing teeth every night or stop eating candy when awake during the night) and thorough descriptions of the specific actions necessary for change were also noted. In addition, possible barriers against being able to change behavior were identified and written down together with strategies for coping with setbacks. The prisoners were provided with the change plan and encouraged to take it back to their own rooms. In line with the MI framework, the intervention ended with a verbal *summary* to make sure the dental hygienist and the prisoners had a common understanding of the plan and to promote optimism (Miller and Rollnick, 2002).

Because of practical reasons and strict prison regulations for conducting research, only one MI-based session (lasting approximately 45 min) was offered. Even though MI is normally provided in more than one single session, several studies indicate that it can initiate behavioral change after only a few freestanding interventions (one to two MI sessions) and that the behavioral change seems to last over time (Antiss *et al.*, 2011; Berman *et al.*, 2010; Rollnick and Allison, 2004; Stenman *et al.*, 2012; Vasilaki *et al.*, 2006). Antiss *et al.* (2011), for example, found that a brief offending-focused MI intervention had a positive effect on the risk of reconviction in male prisoners, while Berman *et al.* (2010) found positive effects of a single MI session in inpatient drug detoxification.

At the end of the intervention (the oral examination and MI-based intervention), the prisoners received the prophylaxis package containing various hygiene aids. This was to ensure that the prisoners were able to perform the oral health-promoting behavior that was decided upon in the MI-based session.

### Data collection method

Given the very limited research on prisoners’ oral health and the lack of knowledge about how to develop successful oral health-promoting interventions directed at this group, a small-scale study with a qualitative methodological approach was adopted (Creswell, 2009). A small-scale or pilot study is especially recommended for areas with limited research and experiences in a given context (vanTeijlingen *et al.*, 2001). A pilot study makes it possible to test interventions on a small scale and make necessary adjustments to adapt to the target population and context in which the study is taking place (Creswell, 2009; Johanson and Brooks, 2010; vanTeijlingen *et al.*, 2001). Qualitative research methods are recommended when the objective is to explore and understand a condition, situation or experience from a personal perspective (Hammarberg *et al.*, 2016) and are considered especially useful for the investigation of patients’ experiences of therapy (Binder *et al.*, 2012). Thematic analysis is one such qualitative approach. It can be considered a foundational method for qualitative analysis, with its focus on detecting, analyzing and reporting *themes* within sets of data. Study design, data collection and data analysis were, therefore, guided by thematic analysis (Braun and Clarke, 2006).

Two clinical examinations were conducted: one prior to the MI-based intervention and one-two weeks after the intervention and the first clinical examination. The first oral examination assessed oral health status and hygiene, whereas the second examination assessed oral hygiene only. Clinical data from both of these examinations were recorded in the dental journal system. The last oral examination was followed by a semi-structured interview with one of the researchers to explore possible short-term effects of the intervention. The interview guide was developed by two of the authors and minor adjustments were made as the interviews progressed. The prisoners were asked about their oral health status and oral health-related behavior prior to and after the intervention, possible explanations of their own oral health and use of the hygiene aids offered in the prophylaxis package. They were further asked about the change plan, if they considered it to be well adapted to their needs and if they had used it actively. Finally, they were asked about their experience with study participation. The interviews lasted between 25 and 60 min and were recorded and transcribed verbatim.

### Analysis of interview data

The analysis of data gathered from the semi-structured interviews was guided by thematic analysis (Braun and Clarke, 2006). First, two of the authors read the transcribed interviews separately to become familiar with the data. Then, each of the authors began to systematically analyze data through coding to identify and obtain an underlying sense of patterns in the prisoners’ responses to the intervention. Following this, the patterns or first dimensions were discussed to reach the first-round agreement before preliminary themes were identified. Data were then further systematized by the authors separately to review the coded data more thoroughly into themes. These systematized data were compared. The themes were then refined and condensed to the present configuration of findings.

### Analysis of clinical data

The oral health status that was assessed in the first oral examination was not assumed to change during the 2-week interval, and therefore, it was assessed only in the first oral examination. MPS was measured on both occasions to evaluate if the intervention had an effect on oral hygiene in the two-week period following the MI session and prophylaxis package. Pre- and post-MPS measurements were compared at an individual level and reported.

## Results

### Data from interviews

Three major themes emerged through the analysis process: how the prisoners regarded their own oral health and how they explained their oral health status, motivational change and behavioral change. The themes are presented in Table 1 and explained in the paragraphs that follow. Quotes from the participants are used to contextualize the themes.

#### Theme 1: Perceptions of oral health status and behavior.

Several of the respondents assessed their oral health status as rather poor. They reported having missing teeth, uncovered treatment needs, pain and toothache. Many of them stated that it was difficult to eat food items such as hard bread, apples or carrots because they were afraid of losing or damaging even more teeth. One of them said:

I suspect that many of my teeth are decayed. It is very painful and it affects what I can eat.

Some of them also reported the social consequences of poor oral health in terms of being hesitant to get in touch with other people, taking fewer initiatives to talk to other people or smiling and laughing less when in a social context:

I miss my front tooth. I smile less than I want to and withdraw from other people.

In total, 5 of the 16 respondents reported regular visits to a dentist. The rest of the samples were irregular in their visits. The prisoners explained their irregular dental attendance to be mainly due to poor finances, dental anxiety or substance abuse. They were not asked about the reasons for their dental fear and none of them elaborated on this. Several of them, however, openly recounted how they failed to prioritize dental treatment when not imprisoned, which was mainly due to unstructured lives, substance abuse and poor finances.

#### Theme 2: Motivational change.

The overall impression from the data analysis was that the prisoners generally lacked understanding and knowledge about the relationship between poor nutrition and hygiene and poor oral health. Many of them displayed a knowledge gap in understanding how their lifestyle could affect oral health:

She told me that smoking might cause cancer. That was very useful. I didn’t know that, that the risk was so high.

During the MI session, the prisoners received individually adapted information on how they could improve and maintain their own oral health. The information provided by the dental hygienist was based on findings from the oral examination and information shared by the prisoners during the MI session. Many of them emphasized how important this concrete and personal information had been in understanding how to improve their own oral health. The respondents also emphasized the importance of being allowed to ask questions about things they did not know:

It was nice and helpful. She listened to me and answered all the questions I had regarding my teeth and how to take care of them.

Several of the respondents expressed an increased understanding of the importance of good oral health and how to maintain it after the intervention. Many lacked knowledge about what causes bad oral health and how to improve it:

[…] she told me […] when I talked to her […] she told me about the germs that stay between the teeth after every meal […]. I think she said that this causes caries? I didn’t know anything about what caused caries before […].

The data analysis indicates that the MI session seemed to bridge the knowledge gap concerning what causes poor oral health and how to improve it. The prisoners reported that this increased knowledge helped them understand better, making them reflect on their own oral health and become more motivated to deal with deficiencies. In addition, all respondents indicated that the intervention had given them a feeling of being understood and taken care of. They reported the MI session with the dental hygienist to be open and nonjudgmental, which they described as crucial for their motivation to change behavior. Some respondents also recounted that they had started to think differently about self-care after the intervention, explaining that they had begun to take better care of themselves in terms of paying more attention to their oral health:

After I talked to her, I have changed a lot about the way I take care of myself and my teeth. It’s funny; it’s so easy and still, I haven’t done it before. This was just what I needed.

Participants observed that this was something new for them and that the intervention had given them the necessary push to start thinking differently about themselves and motivated them to change their behavior.

#### Theme 3: Behavioral change.

Several of the prisoners described their former oral health-related routines as arbitrary and insufficient. Some said that they had tried to establish routines from time to time, but had failed. Many said that this was due to either substance abuse or lack of understanding about the importance of taking good care of their teeth:

I have tried to brush my teeth regularly. But, you know, it is not very easy to always remember when you are on drugs […].

In addition to the emphasis on information and increased understanding, the prisoners emphasized the importance of receiving concrete tools to take care of and improve their oral health. Practical advice about which oral health-related behavior to adopt as set out in the *change plan* was regarded as very helpful by all but one of the respondents. Many did not know how many times a day to brush their teeth to maintain good oral health, nor did they know the importance of using toothpicks after eating:

I use the change plan every day. I kind of remember it, but, anyway, it is very nice to have a reminder. I might have missed it without it or not cared.

How significant the plan was for many of the respondents also became clear through statements such as the following:

The change plan is always beside my bed so that I can read it every time I need an update or give myself feedback. It reminds me of how necessary it is that I brush my teeth twice a day […] and I do brush my teeth twice a day now.

The prophylaxis package was highlighted as a decisive factor in adopting the oral health-related behavior decided on in the MI session and change plan. All the respondents described active use of the different tools in the package:

It was very useful […] of course […] these tools really help cleaning your teeth, it motivates you to take better care of your teeth.

and

I clean my teeth more often after I received this package. Before, when I only had my own toothpaste, I didn’t care that much. Now it is much more fun.

Several of the respondents reported being more conscious of the importance of good oral health, which was initiated by the MI session and reinforced by the good feeling of, for example, brushing teeth or using toothpicks after eating:

My teeth feel much cleaner, I brush my teeth better and more often and it is a very good feeling.

All but three respondents had established new routines when it came to oral health-related behavior after the MI session. Many said that getting help in establishing specific routines and receiving tangible suggestions on what oral health-related behavior to adopt was of vital importance in changing their behavior, while several of them attributed this behavioral change to the change plan and prophylaxis package:

I definitely brush my teeth more often now. And I have started using floss after having received the prophylaxis bag. I clean my teeth more often and more thoroughly.

### Results from the clinical examinations

The oral examinations provided a rough estimate of oral health status and hygiene. The assessment of oral health status showed that seven of the prisoners had one or more missing teeth. Nine needed dental treatment for caries or erosion and were referred to a dentist outside the prison for dental treatment. The first assessment of oral hygiene status – measured by the MPS index – showed that 14 prisoners had a good or acceptable MPS status (score 2–4), one had unacceptable oral hygiene (score 5–6) and one had poor oral hygiene (score 7–8). At the second oral examination, 15 prisoners had good or acceptable oral hygiene (score 2–4), whereas one had an unacceptable score (5–6).

## Discussion

The results of this study indicate that prisoners regard their oral health and related behavior as poor. This is supported by clinical data to a large extent; seven of the prisoners in this study had one or more missing teeth and nine were in need of dental treatment. The prisoners’ oral hygiene status in this study was, in most cases, good or acceptable both before and after the MI intervention. The prisoners reported that their oral health status had negative consequences for social participation, nutrition or food intake and caused toothache and pain. They explained that their poor dental status was caused by dental anxiety, financial problems and substance abuse. The results of this study further suggest that a brief MI-based intervention in a correctional setting may have positive effects on both prisoners’ motivation to perform oral health-related behavior and their performance of oral health-related behavior in a 2-week follow-up. The positive motivational and behavioral changes may also indicate that the intervention was adapted to the specific population and context.

The clinical findings of this study are in line with those of former studies: prisoners generally have poorer oral health status than the non-prison population (Akaji and Ashiwaju, 2014; Guarnizo-Herreño *et al.*, 2014; Heidari *et al.*, 2007; Jones *et al.*, 2002; Osborn *et al.*, 2013; Rodrigues *et al.*, 2014; Vainionpää *et al.*, 2017) and poor oral health has negative consequences for general health and well-being (Slade, 1997). The prisoners’ oral hygiene status in this study was graded as good or acceptable both prior to and after the intervention. The more regular lifestyle a prison environment offers may explain their stable oral hygiene status. However, the conditions under which this examination was conducted may have influenced the accuracy of the dental hygienist’s assessment of oral hygiene: lighting conditions were quite poor compared to a regular clinical situation and dental instruments (e.g. dental probe and suction devices) – normally used in assessing oral hygiene – were not available. The results from the oral examinations of oral hygiene in this study should, therefore, be interpreted with caution and future studies would probably benefit from conducting oral examinations under improved conditions.

It is assumed that several factors are at play in the explanation of poor oral health in this group such as an unhealthy lifestyle, substance abuse, poor finances, dental anxiety, a higher risk for having a disadvantaged background and poor health literacy (Arora *et al.*, 2020; Donelle and Hall, 2014; Donnelly *et al.*, 2019; Freeman and Richards, 2019). The findings of this study largely support this; the participants reported an unhealthy and irregular lifestyle outside prison, substance abuse, lack of money and dental anxiety as reasons for not attending dental treatment regularly. The participants in this study also displayed a lack of basic understanding of the relationship between poor nutrition, poor hygiene and oral health status. This may have impaired their ability to take good care of their oral health. Several of them expressed a fundamental lack of knowledge and understanding of what promotes and maintains good oral health, with a concomitant inability to adopt oral health-promoting behavior. Although no studies have been conducted to our knowledge on prisoners’ health literacy, it is well-established that their level of general literacy is low (Young and Weinert, 2013). However, after attending the MI-based treatment with the dental hygienist, many of them reported an increased understanding of the relationship between such health-promoting behavior and oral health status after a 2-week follow-up. Consequently, they reported an increased motivation for taking better care of their oral health. The data analysis revealed that the enhanced motivation also appeared to be a result of increased self-worth – obtained through the feeling of being understood by the dental hygienist – and consequently increased self-care behavior. The participants in this study further described a change in their oral health-related behavior over the 2 week period in terms of, for example, more frequent brushing, active use of toothpicks, a reduction in soda drinking or less snacking. They described the practical approach of this intervention as a decisive factor in their ability to change behavior. Both the change plan and the prophylaxis package were considered very important by almost all the prisoners. The change plan served as a reminder and concretized both the specific aim and the necessary behavioral steps to reach the goal. It also identified possible obstacles and solutions thereto. The prophylaxis package gave the prisoners the necessary equipment to perform the target behavior and reach the goal set.

MI is designed to change motivation and behavior by exploring and eliciting ambivalence toward behavioral changes and strengthening motivation (Miller and Rollnick, 2002). The intervention applied in this study was, however, not standard MI, as it was based on only three of the four techniques that constitute MI. In the study, *reflective listening* was replaced by a *change plan* to ensure adaptation to the target population and correctional setting. This was necessary because reflective listening is a quite complex technique and its suitability was uncertain given the characteristics of both the prisoners and the correctional setting. MI interventions have shown mixed results when performed with prisoners in a correctional setting and it was of utmost importance to ensure the intervention was well adapted to the population and the setting in which it was performed (Cinar, 2016; Stirman, 2012).

The replacing of reflecting listening with a change plan had two major advantages: first, a change plan could be made very concrete in terms of both setting concrete goals and being a physical paper that the prisoners could carry back to their rooms and refer to. Second, it was relatively simple for the dental hygienist to adapt the change plan individually to each prisoner using information collected in the oral examination or open questions stage. For some, the goal was to brush teeth once a day instead of never or seldom, whereas, for others, it was to reduce the use of tobacco, drink less soda or refrain from snacking in the middle of the night. The findings indicate that the introduction of the change plan was successful and that the prisoners used it more or less on a daily basis. The presented MI intervention, including a concrete change plan and necessary oral hygiene aids, could, therefore, be a promising design for an individually adapted intervention to improve prisoners’ oral health. Reflective listening is, however, an important pillar for change in MI (Miller and Rollnick, 2002) and one should, therefore, be cautious when comparing the presented intervention to other standard MI interventions. In addition, including reflective listening in addition to change plans in future studies could potentially fully exploit the possibilities of the MI methodology.

MI therapy is usually provided in several sessions (Miller and Rollnick, 2002); however, only a single session was offered in this study to encourage motivation and cause change. This was done because the main aim of the study was to test how well the intervention was adapted to the population and research context rather than to optimize its efficacy. The results indicate that the respondents experienced a positive effect from the single session and that the intervention was a good fit with the target population. The results of this study are supported in a meta-analytic review on brief MI interventions, where other studies have also considered the effect of short-term MI interventions (Berman *et al.*, 2010; Vasilaki *et al.*, 2006). For the population in this context, it was of particular interest to test the effect of brief interventions because of the common tendency to transfer prisoners across prisons (Kigerl and Hamilton, 2015). Transfers often happen on very short notice and long-term treatment is, therefore, often not possible. However, future studies should investigate whether several sessions have an increased and prolonged effect.

The alterations of the MI methodology were successful and resulted in positive findings in the study. However, due to the short period between the intervention and the measurement of effect, one should be cautious when drawing conclusions about the longevity impact of the intervention. The reason for the short period between pre- and post-testing was to measure the prisoners’ immediate response to the intervention. The rationale was that their motivation to change briefly after the intervention would reflect whether or not the intervention was adaptable to the specific population and context. However, although the short-term effects of the intervention in this study are positive, one should be cautious to draw conclusions about the long-term effects of the intervention performed in this study. Future research should aim to extend the period between the intervention and the assessment of effect to gain more knowledge on longer-term effect.

One of the characteristics of a pilot study is the low number of participants (vanTeijlingen *et al.*, 2001). All prisoners imprisoned during a given period were asked to participate and all accepted. In total, 7 were transferred or released before the research interview and data from 16 participants were included. Even though data saturation was considered met, one should be cautious when generalizing the results of this study. Nevertheless, the goal of a pilot study is not necessarily to provide conclusive and generalizable answers to research questions but to explore the research topic and research context to be able to adapt an intervention or design a successful large-scale study (vanTeijlingen *et al.*, 2001). This study succeeded in providing findings and experiences that make it possible to design a large-scale study adapted to the target population and adjusted to the prison context.

The study took place in the rigid context of a correctional setting. It is well known that participation in a study may itself affect its results (McCambridge *et al.*, 2014) and awareness of having one’s behavior assessed may prompt beliefs about researchers’ expectations and cause behavior – whether deliberately or not – to change in line with the researchers’ assumed expectations (McCambridge *et al.*, 2011). This participation effect is found in many studies outside a correctional setting and there is no reason to believe that such an effect will decrease in a correctional setting. Participating in a research project while imprisoned will probably represent a break from the tightly regulated and monitored life of incarceration and may well influence the effects of an intervention. In addition, the participants in this study received a gift, which may have contributed even more to a positive attitude toward the intervention and elicited an extra effort to contribute to a positive result. This renders it difficult to conclude which parts of the interventions had the most profound effect on motivational and behavioral changes: the MI session, change plan, prophylaxis package or mere study participation.

Despite the small-scale character of this study, its findings are important because they show clear, positive short-term effects on both motivational and behavioral changes in prisoners’ oral health using sparse resources: a brief oral examination conducted in a visitors’ room in prison, a single-session MI-based intervention with a dental hygienist, a written change plan and a prophylaxis package containing basic aids for oral health care. The findings, therefore, show that it is possible to make difference and bring about change in this vulnerable group in a regulated setting, without large resources or extensive efforts.

One of the aims of this study was to develop an intervention adapted to the specific population and context. The positive findings concerning the prisoners’ responses to the intervention might indicate that the intervention was well adapted and designed to meet their individual needs and preferences. Large-scale studies are, however, needed to refine the intervention and test the long-term impact of the intervention.

## Acknowledgement

This paper and the research behind it would not have been possible without the cooperation of the Norwegian Correctional Service (NCS), who provided necessary insights into the correctional setting and inmate population and facilitated the research project in this setting. The project could not have been completed without the prisoners’ willingness to participate and contribute with their valuable insights and experiences. Also, conducting a research project in a correctional setting is demanding and we are most thankful for all the practical help we received from the staff in the prisons. The Regional Research Funds – West, Norway, provided financial support, and thus made this project possible to conduct.

## Figures and Tables

**Table 1:** Themes, categories and dimensions

Themes	Category	Dimensions
Perception of oral health status and behavior	Social and nutritional consequences	Assessment of own oral health status
		Consequences of oral health status
	Former experiences	Explanations of oral health status
Motivational change	Closing the knowledge gap	New knowledge
		Increased understanding
	Increased self-worth	Feeling of being understood
		Taking care of oneself
Behavioral change	New routines	Practical advice
		Practical tools
